# Losing a Child with HLHS and Creating Brighter Futures for Children and Families

**DOI:** 10.3390/jcdd9050146

**Published:** 2022-05-05

**Authors:** Jessica M. Lindberg

**Affiliations:** Independent Researcher, Caledonia, IL 61011, USA; jessica@ethanlindberg.com

It was a lazy Saturday and my new husband, and I sat on the couch in our Chicago loft to watch TV. We stumbled upon a movie called “Something the Lord Made”, a made-for-TV movie about a cardiac surgeon from Johns Hopkins, Dr. Alfred Blalock, who partnered with Vivien Thomas and Dr. Helen Taussig to perform groundbreaking heart surgery on a baby named Eileen Saxon. Eileen was born with a congenital heart defect called Tetralogy of Fallot and was a “Blue Baby” who did not have enough oxygen in her blood to survive, let alone thrive. On 29 November 1944, this trio performed a surgery that allowed Eileen to have more oxygen in her blood and changed the lives of countless children born with cyanotic congenital heart disease.

We had never heard of congenital heart disease and sat in awe of these doctors who changed history for countless children. I vividly recall thinking about Eileen’s parents. For some reason, I wondered how they decided to agree to the surgery on that day. I guessed they were desperate, though I did not fully understand what that meant at the time. Eileen would only live nine months after the surgery. After her, a nine-year-old girl and a six-year-old boy would undergo the same surgery with better outcomes than Eileen.

That day proved to be an eerie foreshadowing of my life. Less than a year later, at 20 weeks pregnant, I would be sitting in the office of a pediatric cardiologist learning that my firstborn son, Ethan, had evolving Hypoplastic Left Heart Syndrome (HLHS). His left ventricle, while still reasonable size, was threatened by a stenotic aortic valve and mitral valve regurgitation. As I lay on the table for a fetal echo, the pediatric cardiologist told us about a new procedure called Fetal Cardiac Intervention, which opened the aortic valve in utero and allowed blood flow to continue through the left heart with the theory that improved blood flow could potentially salvage the left ventricle and valves. It was a new procedure, and there was no understanding of long-term outcomes, but it looked like there was short-term success.

I was desperate to save my son’s life and give him something better than the current prognosis that HLHS brought. We were candidates for this new experimental procedure, one of the first 30 families to agree to it. Though I did not realize it on that February day in 2005, this choice for my son would change everything in our lives.

Ethan was born in May of 2005 and underwent modified Norwood and Glenn surgeries with an additional conduit replacement in between. His atrial septum was left restricted all three times to allow more blood flow to the left heart, and valvuloplasties modified his abnormal valves. He had challenging surgical recoveries, but we kept our eyes on the prize of preserving his left ventricle with the hopes of it contributing to his circulation. Ethan had an early and traditional Fontan with a wide-open atrial septum, and he enjoyed several years of health. I must interject that Ethan was the happiest boy I have known. He taught me more about living than anything else. Ethan’s zest for life, his love of music, and his family and friends were almost too big to contain. It is as if his spirit was more expansive than his little and sick body. His final hospitalization lasted 13 months in a CICU and included a Fontan takedown, a Bi-Ventricular Repair, a couple of mitral valve replacements, and a VAD placement, after which he died. Ultimately Ethan needed a heart transplant that he was not eligible for due to mounting pulmonary hypertension from his Bi-Ventricular Repair and underlying diastolic dysfunction.

We lived 13 months away from home, we leveraged every financial resource we had, our third child was born amidst that year, and we did our very best to maintain jobs and some semblance of our life while we desperately tried to save Ethan’s. After his death, I combed every one of his medical records, spoke to his doctors and nurses, and reconstructed the choices we had made to understand what had happened. I battled intense guilt, anger, tremendous sadness and ultimately intentionally chose to take what I had experienced and make something beautiful out of it. I decided to own my story and choices and help others faced with similar ones.

One day after Ethan died, my husband said, “We received seven years with Ethan because of so many brave families before us”. I did not want to hear that, but I knew he was right. In a full-circle moment, I thought of Eileen Saxon’s parents who agreed to that first “Blue Baby” Surgery and all the children who have survived and thrived because they, out of love, said yes to trying something innovative and hopeful.

I often wonder if families that have come after us understand the value of what they received from Ethan and the great sacrifice my family has made. I know some children have lived because of him. I know children who have been transplanted earlier because of his story. I know doctors who shifted their ideas about treatment and their advice to families because of him. I wonder if we all realize that ultimately, we stand on the brave shoulders of those who have gone before us. Families, who, out of deep love and hope for their children, agreed to something that ultimately did not work. We stand on the shoulders of doctors and nurses who crafted and participated in these surgeries and treatments, embarking on healing that did not go the way they planned. Any “miracle” in science is often paved with the heartbreak of many.

The missing link in Ethan’s case was his underlying diastolic dysfunction. We got so focused on his left ventricle and “growing” it that we didn’t see or understand, at the time, that it was the etiology of his version of HLHS that, no matter what we did or how big his ventricle was, the plan would not work. Why do some children respond well to surgery or treatment and others do not? Understanding the genetics of this disease is so important so we can tailor treatment to maximize the quality and quantity of our children’s lives.

Upon reading my story, you may think I am against experimental surgeries or research. I am not. I consider my family’s contribution to research to be very significant, whether the average heart family has any clue about it or not. And I know I am not the only family who has contributed in this way. We cannot change the future for our children without making these hard and brave decisions. We must push the envelope, think outside the box, and keep asking, “what if?”. I have a quote by General Patton sitting above my desk that says, “If everyone is thinking alike, then someone is not thinking”. So yes, we must forge ahead.

However, we must also hold the tension of other essential ideals. More than Ethan or Eileen and the many children in between were children with cardiac lesions; they were sons and daughters and brothers and sisters and grandchildren and friends. They embodied hope for the future, their soft cheeks and chunky hands embedded in their mothers’ hearts for eternity. They were families who leveraged jobs and futures to care for their children, often battling mental health and PTSD in daily life. They were siblings longing to grow up together and friends who brought a unique energy to their class or playgroup.

We parents choose surgery and treatment because we hope for thriving children, not just ones who survive. I know this same idea drives many of the incredible doctors, nurses, surgeons, and researchers I know. This tension between the personhood of our children and the challenging medical diagnosis they have is a tightrope that is often hard to balance.

Ethan changed our life. I am who I am because he was here. I would do it all over again in one second. The sacrifice and tears do not outweigh the joy of being his mother.

Would I make different choices?

Yes, I would.

Would I still support research and champion innovation?

Absolutely.

We must forge ahead, holding the tension of progress with the humanity of our children and the needs of our families. We need to continue to recommit to this as a team of parents, medical staff, researchers, and hospital leadership.

In closing, I have eleven suggestions for all of us in this community: parents, caregivers, advocates, medical teams, heart center executives, surgeons, nurses, doctors, and researchers. Holding the tension of all these is not easy, but I challenge us to do it because I believe in us. The future is bright.

1. Temper the drive for innovation with the reality of the human experience and cost.
2. Be honest with each other even when it seems impossible.
3. Reframe success and focus on the quality of life over the quantity of life.
4. Just because you can do an intervention does not mean you should.
5. If you are not sure what to do next, phone a friend. Respect and consider other/competing ideas: less ego, more collaboration.
6. Keep asking questions and looking for the slight, obscure evidence and nuance that may be telling you something.
7. Programs are made possible by the love and sacrifice of both the medical team and the families. Great people make great programs. Support your team and families.
8. Understand the genetics of CHD so you can tailor the right surgeries and treatments to the right patients. Fund this research and support researchers on this quest.
9. Be honest about trauma’s role in all of this. Take care of your mental health. Get help if you need it and recognize that medical staff, families, and patients have equally been traumatized by their experience.
10. Our hospital magazines must not only be shiny pictures of success that help to raise more money but also must honor the sacrifices of families and their medical team who came up short and, in turn, will bring healing to many others.
11. Every day is a beautiful gift. Let’s keep moving forward.

Jessica Lindberg is the Founder and President of Ethan Lindberg Foundation and The Heart Strong Collective. To learn more about her work visit www.theheartstrong.com (accessed on 2 May 2022).

